# The continuum of care for maternal health in Africa: A systematic review and meta-analysis

**DOI:** 10.1371/journal.pone.0305780

**Published:** 2024-07-18

**Authors:** Ritbano Ahmed, Solomon Gebre, Minychil Demelash, Tamiru Belachew, Abdurezak Mohammed, Abdulhakim Musema, Mohammed Sultan

**Affiliations:** 1 Department of Midwifery, College of Medicine and Health Sciences, Wachemo University, Hossana, Ethiopia; 2 Department of Laboratory, College of Medicine and Health Sciences, Wachemo University, Hossana, Ethiopia; 3 Department of Statistics, Collage of Natural and Computational Science, Wachemo University, Hosanna, Ethiopia; African Population and Health Research Center, ETHIOPIA

## Abstract

**Background:**

The continuum of care for maternal health (COCM) is a critical strategy for addressing preventable causes of maternal and perinatal mortality. Despite notable progress in reducing maternal and infant deaths globally, the problem persists, particularly in low-resource settings. Additionally, significant disparities in the provision of continuous care exist both between continents and within countries on the same continent. This study aimed to assess the pooled prevalence of completion across the maternity care continuum in Africa and investigate the associated factors.

**Methods:**

Relevant articles were accessed through the EMBASE, CINAHL, Cochrane Library, PubMed, HINARI, and Google Scholar databases. Funnel plots and Egger’s test were employed to assess publication bias, while the I-squared test was used to evaluate study heterogeneity. The inclusion criteria were limited to observational studies conducted exclusively in Africa. The quality of these studies was assessed using the JBI checklist. Data extraction from the included studies was performed using Microsoft Excel and then analysed using Stata 16 software.

**Results:**

A total of 23 studies involving 74,880 mothers met the inclusion criteria. The overall prevalence of women who successfully completed the COCM was 20.9% [95% CI: 16.9–25.0]. Our analysis revealed several factors associated with this outcome, including urban residency [OR: 2.3; 95% CI: 1.6–3.2], the highest wealth index level [OR: 2.1; 95% CI: 1.4–3.0], primiparous status [OR: 1.3; 95% CI: 2.2–5.1], planned pregnancy [OR: 3.0; 95% CI: 2.3–3.7], and exposure to mass media [OR: 2.7; 95% CI: 1.9–3.8].

**Conclusion:**

The study revealed that only 20.9% of women fully completed the COCM. It also identified several factors associated with completion of the COCM, such as residing in urban areas, possessing a higher wealth index, being a first-time mother, experiencing a planned pregnancy, and having access to mass media. Based on the study’s findings, it is recommended that targeted interventions be implemented in rural areas, financial assistance be provided to women with lower wealth index levels, educational campaigns be conducted through mass media, early antenatal care be promoted, and family planning services be strengthened.

**Review registration:**

PROSPERO International Prospective Register of Systematic Reviews (ID: CRD42020205736).

## Introduction

The maternity care continuum encompasses the full spectrum of care provided to women throughout their pregnancy, childbirth, and postpartum periods [1]. This comprehensive service is designed to enhance public awareness and encourage women to access maternal healthcare services. Integrating interventions in this manner is vital as it can lead to cost reductions by enhancing efficiency, uptake, and promotion of other healthcare components [1, 2].

Maternal deaths, particularly those that are preventable, continue to be a significant issue in Africa [3, 4]. These tragic events typically occur during three specific time frames: prenatal, intrapartum, and postpartum [4]. In 2020, a total of 287,000 global maternal deaths were reported, with the majority (94%) occurring in low- and middle-income countries, despite the availability of simple, low-tech interventions in these regions. Sub-Saharan Africa alone accounted for approximately 70% of maternal deaths (202,000) [5]. Maintaining care from pregnancy to childbirth and during the postnatal period significantly reduces the unacceptably high rates of maternal and child mortality and morbidity [1, 4, 6–10]. This service offers a substantial opportunity to provide fully integrated care delivery, ensuring routine monitoring and a consistent opportunity for critical intervention throughout pregnancy, the crucial months post-delivery, and the entire postnatal period [10–13].

Furthermore, the complete utilization of all components of the continuum of care (COCM) guarantees access to essential healthcare services, including lactation support, chronic condition management, and postpartum mental health screenings [10]. It also enhances the completion rate of infant immunization and postnatal family planning uptake [14, 15].

In Africa, the completion rate and associated factors of the COCM vary by country [16–23], with no single figure identified. Moreover, different definitions have been used in studies on this topic [19–29]. Addressing these issues is crucial for the development of effective initiatives to improve maternal healthcare utilization in Africa. Systematic review and meta-analysis designs are suitable for tackling these challenges. The aim of this study was to determine the prevalence of the completion of the COCM and its associated factors in Africa.

## Methods

The systematic review and meta-analysis were conducted in accordance with the Preferred Reporting Items for Systematic Reviews and Meta-Analyses (PRISMA) guidelines [30] (**S1 File**). The eligibility criteria were adapted from the JBI 2017 review guidelines [31]. The review protocol was developed following PRISMA 2020 guidelines, submitted, and subsequently published in PROSPERO International’s prospective register of systematic reviews (ID: CRD42020205736). Endnote (version X8) reference management software was used to download, organize, and review references, while Zoster was utilized to cite relevant articles.

### Search strategy

Six databases, namely PubMed, Hinari, EMBASE, Cochrane, CINHAL, Google Scholar, and Mednar, were systematically searched for published articles related to Africa. In addition, unpublished articles were sought from the institutional repositories of Addis Ababa University, Mekele University, and Jimma University. The search strategy involved querying the PubMed and Google Scholar databases using article titles to identify relevant key terms, which were further explored in the articles’ abstracts, keyword lists, and indexes. The search terms used encompassed a range of terms such as "maternity continuum of care," "continuum," "maternity care," "associated factors," "continuum of care," "continuum of care for maternal health services," "maternal health," "level," "coverage," "magnitude," "associated factors," "mother," "women," "determinants," "predictors," and "Africa." These search terms were combined using Boolean operators such as “OR” for similar terms and “AND” for different concepts. The search included articles published in English between September 28, 2015, and September 28, 2022 (**S2 File**).

### Inclusion criteria

#### Study area

Africa.

#### Study participants

This review included all studies that examined the completion level of the maternity continuum of care and its associated factors. The participants in these studies were women who had at least one birth.

#### Types of studies

The studies included in this review were observational in nature.

#### Publication conditions

Both published articles and gray literature were considered.

#### Language

Only English language studies were included, provided that they contained the minimum information required to calculate a pooled analysis of prevalence. This information included the number of subjects and the number of completions on the maternity care continuum.

#### Outcome of interest

The primary focus of this review was to examine the completion rate of the continuum of maternal care and its associated factors.

#### Publication date

The studies considered in this review were conducted between September 28, 2015, and September 28, 2022. This time frame was chosen because there were no relevant studies identified on this topic in Africa prior to 2015.

### Inclusion and exclusion criteria

To ensure the scholarly robustness of our study, we have established a stringent set of inclusion and exclusion criteria for articles. Firstly, articles that were devoid of an abstract and/or full-text were excluded, as these materials are indispensable for a comprehensive analysis. Moreover, we deemed it necessary to exclude anonymous reports and qualitative studies due to their potential deficiency in furnishing the requisite empirical evidence for our research. Furthermore, articles that failed to incorporate events in either the risk or nonrisk groups following a minimum of two email correspondences with the primary author were similarly excluded.

### Outcome measurement

This systematic review encompasses two outcomes. The primary outcome variable of this study is the **completion of a continuum of care**. We defined this based on previous recommendations for focused antenatal care (ANC) because not all African countries have implemented the WHO 2016 ANC model, which advocates for a minimum of 8 ANC visits during pregnancy. In this study, women were considered "complete" if they had received at least four or more antenatal visits (ANC4+), skilled birth attendance (SBA), or at least one postnatal care (PNC) contact. Conversely, women are deemed "incomplete" if they have received ANC4+ and SBA but not PNC. The second outcome measures the associated factors of the continuum of care. For the calculation of the pooled odds ratio, we considered primary studies that examined the relationship between each factor and the continuum of care. The classification or definition of each independent or associated factor was as follows: place of residence was classified as rural or urban, maternal age was grouped as 25–34 or other (below 25 or above 35) years old, and women’s educational status was categorized as secondary education and above or other. Parity was categorized as primiparous (for first-time mothers) or other (including multi and grand-multiparous). Pregnancy status was divided into intended or unintended (missed time or unwanted). Exposure to mass media was classified as "yes" (exposure to at least one form of media) or "no." Wealth status was classified into two categories: lowest (poor/poorest) and highest (rich/richest). Women’s autonomy was categorized as either autonomous (the woman alone making maternal care-seeking decisions) or not autonomous (the decision made by the husband alone or as a joint decision). Occupational status was categorized as working outside the home or working inside the home. The timely initiation of ANC was defined as the first ANC visit occurring at or before 16 weeks of gestation. Distance from the nearest health facility was grouped as near or far, using estimated distance or travel time from the nearest health facility. If the estimated distance is greater than 5 km or the travel time is less than 30 minutes by car, it is classified as near; otherwise, it is considered far. Knowledge of key pregnancy danger signs: In this study, we considered a woman "knowledgeable" if she spontaneously mentioned at least two key danger signs of pregnancy (vaginal bleeding, severe headache, blurring of vision, and swelling of feet or face); if not, she was classified as "unknowledgeable." Birth preparedness and complication readiness (BPCR): We classified women as "well prepared" for birth and its complications if they reported implementing five or more components of birth preparedness and complication readiness (BPCR); otherwise, they were considered "not well prepared."

### Data extraction

A standardized data extraction format was employed to collect the essential information required for analysis. The extraction form comprised the following elements: the author’s last name, publication year, geographical location of the study, study design, data source, sample size, confidence interval for the completion rate of the maternity care continuum, and the quality score for each study. Two authors independently conducted the data extraction process, cross-validated the data, and resolved any discrepancies through consensus. Quantitative data were extracted using Microsoft Excel (**S3 and S4 Files**).

### Quality of studies

The Joanna Briggs Institute (JBI) quality appraisal checklist [31] was utilized to evaluate the quality of individual studies. Two authors independently assessed the quality of each primary study, and any discrepancies between the two authors’ assessments were resolved by averaging their scores. A study was categorized as having a "low risk" if it achieved over 50% of the quality assessment indicators. All the identified studies were cross-sectional and were evaluated based on eight criteria: inclusion criteria, description of study subjects and setting, valid and reliable measurement of exposure, objective and standardized criteria, identification of confounders, strategies to address confounders, outcome measurement, and appropriate statistical analysis. Ultimately, a total of 40 studies were evaluated, out of which 23 received a quality score of 50% or higher, indicating that they were considered low risk and therefore included in the analysis. The remaining 16 studies received a high-risk score and were excluded from the analysis (**S5 File**).

### Statistical analysis

The statistical analysis was carried out using Stata statistical software (version 16.0, Statacorp. LP, College Station, United States of America). We employed random effects methods to pool the prevalence of completion of the continuum of care for maternal health in Africa. To identify factors associated with the completion of the maternal health continuum of care, both random and fixed effect models were utilized. Statistical significance was determined using a p-value and a 95% confidence interval. The random effect model was used for analyses with statistical heterogeneity, while the fixed effect model was used for analyses without heterogeneity. Statistical heterogeneity was assessed using the I-squared [I2] statistic test [32]. Subsequently, meta-regression and subgroup analysis were conducted to explore potential sources of heterogeneity. Visual assessment of funnel plot asymmetry and Egger’s test [33] were used to evaluate publication bias. However, as the p-value was > 0.05, there was no statistical evidence for the presence of publication bias according to Egger’s test. Finally, a sensitivity analysis was performed to assess the impact of individual studies on the overall estimate.

## Results

A total of 1,817 studies were identified in the various databases following an initial search. Five hundred nineteen duplicate studies were identified and subsequently removed, leaving 1,196 articles for further evaluation. The titles and abstracts of these 1,196 articles were carefully reviewed, resulting in the exclusion of 1,158 irrelevant investigations based on the title or abstract. Subsequent evaluations of 40 studies for eligibility led to the removal of 17 additional studies. Twelve studies were excluded due to variations in outcome definitions [24–26, 28, 29, 34–40], while five other studies were excluded to avoid duplication of study participants [27, 41–44]. Ultimately, 23 studies that met the inclusion criteria were included in this study [16–23, 45–59] (**S1 Fig**).

### Study characteristics

We analysed 23 studies conducted in nine African countries, including a total of 74,880 study participants. These studies focused on the continuum of care during the participants’ most recent pregnancy. All of the studies included in our analysis were cross-sectional in nature, with varying sample sizes ranging from 289 [58] to 24,502 [16]. The included countries were Ethiopia (14 studies), Tanzania (1 study), Kenya (1 study), Ghana (2 studies), Egypt (1 study), Uganda (1 study), the Democratic Republic of the Congo (1 study), Zambia (1 study), and Guinea (1 study). The completion rate of the continuum of care varied across countries, ranging from 6.9% in Ethiopia [59] to 50.4% in Egypt [50]. **Table 1** presents the characteristics of the included studies.

**Table 1:** Description of included studies in the meta-analysis of the pooled prevalence and associated factors of completion of the COCM in Africa, 2022.

Author and year	Country	Source of data	Region	Sample	Frequency	Prevalence [95% CI]
Shibanum et al., 2018 [20]	Ghana	Primary	West Africa	1401	357	25.5[23.3, 27.8]
Hamed et al., 2018 [50]	Egypt	Primary	North Africa	2790	1406	50.4[48.5,52.2]
Ahmed et al., 2022 [53]	Ethiopia	Primary	East Africa	816	92	11.3[9.3, 13.6]
Abebe et al., 2022 [55]	Ethiopia	Secondary	East Africa	2905	375	12.9[11.7, 14.2]
Emiru et al., 2020 [21]	Ethiopia	Primary	East Africa	1281	155	12.1 [10.4, 14.0]
Atnafu et al., 2020 [49]	Ethiopia	Primary	East Africa	565	122	21.6 [18.4, 25.2]
Arunda et al 2021 [16]	Kenya	Secondary	East Africa	24502	4961	20.3[19.7,20.8]
Camara et al., 2021 [22]	Guinea	Secondary	West Africa	3018	604	20.0 [18.6, 21.5]
Chaka 2022 [51]	Ethiopia	Secondary	East Africa	7950	731	9.2 [8.6,9.8]
Cherie et al., 2021 [54]	Ethiopia	Primary	East Africa	732	82	11.2 [9.1, 13.7]
Dadi et al., 2022 [47]	Ethiopia	Primary	East Africa	1431	199	13.9 [12.2, 15.8]
Desta et al., 2021 [57]	Ethiopia	Primary	East Africa	585	199	34.0 [30.3, 37.9]
Haile et al., 2019 [52]	Ethiopia	Primary	East Africa	432	42	9.7 [7.3, 12.9]
H/Mariam et al., 2022 [59]	Ethiopia	Primary	East Africa	811	56	6.9[5.4, 8.9]
Mohan et al., 2017 [23]	Tanzania	Primary	East Africa	1931	198	10.3 [9.0, 11.7]
Sertsewold et al., 2021 [18]	Ethiopia	Primary	East Africa	514	99	19.3 [16.1, 22.9]
Sserwanja et al., 2021 [48]	Zambia	Secondary	East Africa	7325	2787	38.0 [36.9, 39.2]
Sserwanja et al., 2022 [45]	Uganda	Secondary	East Africa	10152	1092	10.8 [10.2, 11.4]
Tiruneh et al., 2022[56]	Ethiopia	Primary	East Africa	2724	586	21.5 [20.0, 23.1]
Tizazu et al., 2021[19]	Ethiopia	Primary	East Africa	647	241	37.2 [33.6, 41.0]
Yeji et al., 2015 [46]	Ghana	Primary	West Africa	1497	376	25.1[23.0, 27.4]
Tadese et al., 2022 [17]	Ethiopia	Primary	East Africa	842	260	30.9 [27.85,34.08]
Galle et al., 2022 [58]	DR Congo	Primary	Middle Africa	289	104	36.0 [30.67,41.67]

### Pooled prevalence of completion of the continuum of care

This study revealed that the overall prevalence of the continuum of care in Africa, determined using a fixed effects model, was 16.9% (95% CI: 16.67–17.20). The heterogeneity test for the proportion of the reviews showed an I2 value of 99.5% and a P value of 0.00, indicating significant variation among the included studies. To account for this heterogeneity, a random-effects model was used. With the random-effects model, the final pooled prevalence of the continuum of care in Africa was estimated to be 20.9 (95% CI: 16.9–25.0). It is important to note that the prevalence of the continuum of care varied across the different studies. The overall pooled prevalence of the continuum of care is shown in **S2 Fig**.

#### Subgroup analysis by region and country

We conducted subgroup analysis to address heterogeneity, taking into account the data source and regional and country variations. The subgroup analysis revealed varying prevalence rates of completing the continuum of maternal health care across different regions of Africa. Specifically, the prevalence rates were 50.4%, 18.1%, 23.3%, and 35.9% in the Northern, Eastern, Western, and Middle Africa studies, respectively. The completion rate for all components of maternity care ranged from 50.4% in Egypt to 10.25% in Tanzania (**Table 2**). Notably, no studies from the southern African region were included. Furthermore, we conducted regression analysis using sample size and publication year as variables to explore potential sources of heterogeneity. The results indicated that heterogeneity could not be explained by sample size (P  =  0.824) or publication year (P  =  0.385) (**Table 3**).

**Table 2:** Subgroup analysis of the pooled prevalence of completion of the COCM in Africa, 2022.

Variable	Characteristics	Pooled prevalence 95%CI	I^2^	P value
**By region**	East Africa	17.3% [13.3, 21.4]	99.6%	0.00
	West Africa	23.3% [19.1, 27.8]	--	--
	North Africa	50.4% [48.5, 54.3]	--	--
	Middle east	36.0% [30.7, 41.7]	--	--
**Country**	Ethiopia	17.5% [14.0, 21.1]	98.2%	0.00
	Kenya	20.3% [19.8, 20.7]		
	Ghana	25.3% [23.7, 26.9]	--	--
	Guinea	19.6% [18.2, 21.0]	--	--
	Uganda	10.8% [10.2, 11.4]	--	--
	Tanzania	10.3% [9.0, 11.2]	--	--
	Zambia	38.1% [37.0, 39.2]	--	--
	Egypt	50.4% [48.5, 52.3]	--	--
	DR Congo	36.0% [30.7, 41.7]	--	--

**Table 3:** Meta-regression analysis of factors affecting between-study heterogeneity.

Source of heterogeneity	Coef	Std. Err.	t	P>t	[95% Conf. Interval]	
Sample size	-.000107	.0004809	0.22	0.824	--.0010496	.0008356
Publication year	-1.007256	1.159994	-0.87	0.385	-3.280803	1.266292

### Publication bias

To evaluate publication bias, we utilized either a funnel plot or Egger’s regression test. Upon subjective evaluation of the funnel plot **(S3 Fig)**, we observed a symmetrical distribution. The p value from Egger’s regression test was 0.41, suggesting the absence of publication bias. However, we did not conduct the Duval and Tweedie nonparametric "trim and fill" analysis to identify potential publication bias.

### Sensitivity analysis

A sensitivity analysis was conducted to assess the impact of each individual study on the overall meta-analysis summary estimate. None of the studies had a significant effect on the final pooled prevalence of maternal health care completion (**Table 4**).

**Table 4:** Sensitivity analysis for the effect of the COCM in Africa, 2022.

Study omitted	Estimate	95% Conf. Interval]	
Hamed et al., 2018 [50]	19.55	16.01	23.10
Ahmed et al., 2022 [53]	21.38	17.24	25.51
Abebe et al., 2022 [55]	21.31	17.10	25.52
Emiru et al., 2020 [21]	21.34	17.19	25.49
Arunda et al., 2021 [16]	20.99	16.34	25.64
Atnafu et al., 2020 [49]	20.91	16.79	25.02
Camara et al., 2021 [22]	21.00	16.81	25.20
Chaka 2022 [51]	21.49	17.28	25.69
Cherie et al., 2021 [54]	21.38	17.25	25.51
Dadi et al., 2022 [47]	21.26	17.10	25.42
Dasta et al., 2021 [57]	20.35	16.27	24.44
Haile et al., 2020 [52]	21.44	17.32	25.56
H/mariam et al., 2022 [59]	21.58	17.47	25.69
Mohan et al., 2017 [23]	21.43	17.26	25.59
Sertsewold et al., 2021 [18]	21.15	17.03	25.28
Sserwanja et al., 2021 [48]	20.11	16.59	23.64
Sserwanja et al., 2022 [45]	21.41	17.06	25.77
Tiruneh et al., 2022 [56]	20.95	16.77	25.13
Tizazu et al., 2021 [19]	20.21	16.14	24.28
Shibamun et al., 2018 [20]	20.73	16.61	24.85
Yeji et al., 2015 [46]	20.75	16.62	24.87
Tadese et al., 2022 [17]	20.49	16.40	24.58
Galle et al., 2022 [58]	20.30	16.21	24.38
**Combined**	**20.94**	**16.92**	**24.95**

### Factors associated with the completion of the COCM in Africa

The variables included residence (urban), education level (secondary and above), wealth index (highest), parity (primipara), first ANC visit (on time), pregnancy status (wanted), distance from the nearest health facility (near), and birth and complication readiness plan (well planned). The findings showed that women residing in urban areas were approximately two times more likely to utilize the entire COCM compared to women living in rural areas [OR = 2.3; 95% CI: 1.6–3.2]. Moreover, the completion rate of the maternity care continuum was highest among women who had achieved a secondary education or higher [OR = 2.0; 95% CI: 1.5–2.7]. Furthermore, the probability of completing the continuum of maternity care was 110% higher among women from families with the highest economic status [OR = 2.1; 95% CI: 1.4–3.0]. Additionally, it was found that women who had an early first ANC visit or a planned pregnancy, as well as primiparous women, were 3.4, 3.0, and 1.3 times more likely, respectively, to receive a full COCM compared to their counterparts. Furthermore, women residing near health facilities were 3.4 times more likely to utilize a complete COCM than those living far from health facilities. Moreover, women who had a history of mass media exposure were approximately 2.5 times more likely to complete the continuum of maternity care than those who had no exposure. Additionally, women who had well-prepared birth preparedness and complication readiness plans were approximately 120% more likely to complete the continuum of maternity care than those who were not prepared. More details regarding the associated factors can be found in **Table 5** and **S4–S16 Figs**.

**Table 5:** Factors associated with completion of the COCM in Africa, 2022.

Factors	Comparison	NS^a^	SS^b^	OR (95% CI)	I^2^%	Egger test (P–value)	Figures
Residence [16, 17, 22, 45, 48, 50, 51, 53–55]	Urban Vs Rural	10	60,666	2.3[1.6,3.2] *	97.8	0.13	S4 Fig
Age [16–19, 22, 45, 48, 51, 53, 55, 59]	25–34 years Vs others	11	59,182	1.2[1.00,1.3]	83.8	0.2	S5 Fig
Education [17–19, 45, 48, 49, 51–53, 55, 56, 58, 59]	Secondary-above Vs others	13	35,431	2.0[1.5,2.7] *	94.0	0.7	S6 Fig
Occupation [17, 45, 48–50, 52, 53, 58]	Employed vs nonemployed	8	32,262	1.4[0.4,2.3]	99.7	0.4	S7 Fig
Wealth index [22, 45, 48, 50, 53, 55, 56, 59]	Highest Vs lowest	8	30,641	2.1[1.4, 3.0] *	96.8	0.8	S8 Fig
Parity [18, 48, 50, 52, 53, 55]	Primipara Vs others	7	25,280	1.3[1.1, 1.5] *	68.0	0.3	S9 Fig
First ANC visit [18, 45, 48, 52, 53, 55]	Timely vs Late	6	21893	3.4[2.2,5.1] *	95.5	1.0	S10 Fig
Planned pregnancy [17, 18, 52–54, 57, 59]	Yes Vs No	7	4,832	3.0[2.3,3.7] *	0.0	0.4	S11 Fig
Distance from health facilities [18, 49, 52, 53, 56, 59]	Near vs Far	6	5866	3.4[1.4,8.2] *	95.8	0.7	S12 Fig
Mass media exposure [17, 18, 45, 48, 50, 53, 54]	Yes Vs No	8	22,934	2.7[1.9,3.8] *	92.9	0.5	S13 Fig
Knowledge regarding pregnancy danger signs [19, 52, 53]	Knowledgeable vs Not	3	1895	1.9[0.9,4.1]	85.3	0.5	S14 Fig
BPCRP [19, 52, 53, 57]	Well-prepared Vs not prepared	4	2480	2.2[1.8,2.7] *	0.0	0.9	S15 Fig
Autonomy [18, 53, 54]	Autonomous Vs not-autonomous	3	2144	10.0[3.3,30.2] *	89.7	0.03	S16 Fig

^a^ Number of studies

^b^ Sample size

* P value<0.05

## Discussion

The pooled prevalence of continuum of care in Africa was found to be 20.9%. This figure is lower than the prevalence reported in studies conducted in India (39%, 17%) [60, 61], Bangladesh (30.5%) [62], Nepal (45.7%, 41%, and 75%) [62–64], Pakistan (27%) [65], and Cambodia (60%) [66]. The lower prevalence in Africa may be attributed to limited access to maternal healthcare services. Additionally, this lower value may be due to the fact that while previous studies defined the continuum of maternal health care as the proportion of women who had any antenatal care (ANC) visit to at least one postnatal care (PNC) visit, our analysis focused specifically on studies that defined the continuum of maternal health care as the proportion of women who had at least one ANC4+ visit to at least one PNC visit within the postnatal period. However, it is important to note that this prevalence rate is higher than those reported in studies conducted in Cambodia (5%) [67] and the Lao People’s Democratic Republic (LPDR) (6.8%) [68].

These differences in prevalence rates may be attributed to sociocultural factors. In order to achieve the health targets outlined in Sustainable Development Goal 3, it is crucial to improve the continuum of maternity care across the African continent. Implementing various life-saving measures at every stage of care, including prenatal, intrapartum, and postnatal care, is essential. Governments of African countries should prioritize the expansion of coverage for COCM services and ensure adequate follow-up for antenatal care visits. Furthermore, these findings suggest that the most effective maternal healthcare services may not be uniformly implemented across the continent. Enhancing the completion rate of the maternity care continuum is likely to have a positive impact on maternal health. Strategies such as incorporating counseling on the components of the maternity care continuum during initial and subsequent antenatal care appointments should be implemented.

The completion rate of the maternity care continuum varies significantly across different geographic regions and countries in Africa, ranging from 18.1% in the east to 50.4% in the north. This variation can be attributed to socioeconomic and cultural factors, as well as the implementation of interventions for women. These findings present a significant opportunity for the continent. We evaluated this issue based on the previously recommended ANC guidelines, which are relatively easier to achieve in low-resource countries compared to the current WHO ANC recommendation. To meet the 2016 WHO recommendation, all African governments must make concerted efforts and implement effective strategies.

Women with a secondary education or higher are more likely to utilize the complete continuum of care for maternal health. This assertion is substantiated by studies conducted in numerous countries including South Asia [41], India [61], Nepal [63], Pakistan [65], and the LPDR [68]. Educated women are more knowledgeable about the maternal healthcare services that are available and understand the advantages of utilizing all aspects of the maternity continuum of care to optimize the health of both mothers and infants. It is imperative to enhance maternal education in order to increase the utilization of maternal healthcare services.

The utilization of the complete continuum of care for maternal health is higher among women living in urban areas compared to those residing in rural areas. This could be attributed to better access to healthcare services and information concerning all aspects of maternal care in urban areas. Consequently, women in urban areas engage with the entire spectrum of maternal health services. Studies conducted in South Asia [41], India [60, 61], Nepal [62–64], and Pakistan [65] validate this analysis and underscore the greater utilization of the continuum of care by women in urban areas. Particular attention should be given to improving access to maternal healthcare services in rural areas in order to achieve target levels of care for these women.

The timing of the first antenatal care (ANC) visit is linked to the completion of the maternity care continuum as indicated by this meta-analysis. Early initiation of ANC presents an opportunity for women to be informed about all available maternal healthcare services from the pregnancy period through the postnatal period. Additionally, early initiation of ANC enables healthcare providers to counsel women on the recommended components and benefits of receiving all aspects of maternity care for maternal and fetal health, resulting in a higher likelihood of completing the continuum of care. The findings from research conducted in the LPDR support this notion [68].

Furthermore, this meta-analysis demonstrated that women who plan their pregnancies are more likely to complete the maternity care continuum compared to those who have unplanned pregnancies. This finding suggests that women who actively plan their pregnancies have greater aspirations for positive pregnancy, birth, and postnatal experiences, as well as favourable birth outcomes. Consequently, these women are more likely to remain engaged in the continuum of maternal healthcare. Studies conducted in India [61] and Bangladesh [69] also validate this finding. Primiparity was found to be significantly associated with the complete utilization of the maternal continuum of care. This finding aligns with previous research conducted in South Asia [41], Nepal [62, 70], Pakistan [65], and Cambodia [66]. One potential explanation for this is that women who have not previously experienced the pregnancy-to-postpartum process might have a stronger desire to understand their health status and access available care throughout each stage.

In fact, individuals who possess more information about specific services are more likely to utilize them compared to those who have received no information. This meta-analysis demonstrates that women who have been exposed to mass media are more inclined to utilize all components of the maternal health care continuum, consistent with research conducted in India [63], Nepal [62–64], and Pakistan [65].

Furthermore, this study reveals that women living near health care facilities are more inclined to utilize all facets of maternity care. One possible explanation for this phenomenon is that geographic proximity to these facilities encourages women to seek care in order to avoid transportation-related challenges and delays. This finding aligns with research conducted in Nepal [64], Cambodia [67], and LPDR [68].

According to this meta-analysis, having the highest household wealth status is strongly associated with the utilization of the entire continuum of care for maternal health. This finding is supported by research conducted in various countries, including South Asia [41], India [61], Bangladesh [69], Nepal [62–64], and Pakistan [65]. These studies highlight that women belonging to the highest household wealth quintile are more likely to consistently access maternal care. Additionally, individuals with higher socioeconomic status tend to utilize more healthcare services, a trend supported by numerous studies.

Completing the maternal care pathway has been found to be associated with women’s autonomy in making healthcare decisions. Consistently, studies conducted in Pakistan [65], LPDR [68], and Nepal [70] have demonstrated a significant association between women’s autonomy and the utilization of all components of the maternal health care continuum. This suggests that women who have full independence are able to utilize maternal healthcare services without external interference, prioritizing maternal health care.

Moreover, women who have a well-planned birth and a complication readiness plan are more likely to complete the continuum of care for maternal health, consistent with other studies [52, 53]. This could be partly explained by the fact that as women become better prepared, their confidence in accessing and utilizing maternal health care services increases. Therefore, women who have completed all components of maternal care are more likely to receive the necessary maternal health care.

## Limitations of the study

The study has several limitations that need to be considered. Firstly, all the studies included in the review were cross-sectional, which may introduce recall bias, potentially leading to an underestimation or overestimation of the outcome variable. Moreover, some studies lacked a sufficient number of predictor variables, thereby limiting their ability to make accurate predictions. Another limitation is the lack of representation from the southern regions of the African continent in the meta-analysis. However, it is worth noting that the demographic characteristics of the South African population were similar to those of other regions. Additionally, certain variables related to neonatal sepsis, identified in various primary articles across Africa, were excluded either because they were present in only one article or because their classification differed from that of the included articles. It is crucial to recognize that meta-analyses of prevalence often exhibit significant variation, which can be attributed to differences in the time and location of the studies included. Therefore, caution is advised when interpreting these findings. Furthermore, it should be highlighted that *I*^2^ estimates may not always be reliable due to limited power and precision. High heterogeneity could be influenced by factors such as time-dependent bias or sample size dependence.

## Conclusion

This meta-analysis highlights the significantly low proportion of patients who completed the COCM for maternity health in Africa, with a prevalence of 20.9%. Factors such as urban residency, higher wealth index, primiparous status, planned pregnancy, and exposure to mass media are significantly associated with a greater likelihood of completing the COCM. Both governmental and nongovernmental organizations involved in improving maternal health in Africa should prioritize the implementation of targeted interventions to enhance access to maternal health services in rural areas. This will help bridge the gap between urban and rural areas in terms of completing the COCM. Furthermore, providing financial support or incentives to women from lower socioeconomic backgrounds is crucial to ensuring that maternal health services are affordable for everyone. Education campaigns should be conducted through mass media channels to raise awareness about the importance of maternal health care and the benefits of completing the COC. Additionally, promoting early antenatal care visits is essential to ensuring the completion of maternal healthcare services. It is also recommended to strengthen family planning services to support planned pregnancies, as they have shown higher rates of completing the COC.

## Supporting information

S1 File PRISMA 2020 checklist. (DOCX)

S2 File Search terms used in each database. (DOCX)

S3 File Data extraction form for prevalence. (XLSX)

S4 File Data extraction form for factors. (XLSX)

S5 File Quality appraisal of included study. (DOCX)

S1 Fig RISMA flowchart diagram flow diagram of the study selection process. (TIF)

S2 Fig Pooled prevalence of completion of COCM in African, 2022. (TIF)

S3 Fig Funnel plot for publication bias for prevalence of completion of the COCM in Africa, 2022. (TIF)

S4 Fig The pooled odds ratio for the association between residence and completion of COCM in Africa, 2022. (TIF)

S5 Fig The pooled odds ratio of the association between maternal age and the completion of the COCM n Africa, 2022. (TIF)

S6 Fig The pooled odds ratio of the association between maternal education and the completion of the COCM in Africa, 2022. (TIF)

S7 Fig The pooled odds ratio of the association between maternal employment status and the completion of the COCM Africa, 2022. (TIF)

S8 Fig The pooled odds ratio of the association between wealth index and the completion of the COCM Africa, 2022. (TIF)

S9 Fig The pooled odds ratio of the association between parity and the completion of the COCM Africa, 2022. (TIF)

S10 Fig The pooled odds ratio of the association between timely initiation of ANC visit and the completion of the COCM Africa, 2022. (TIF)

S11 Fig The pooled odds ratio of the association between pregnancy status and the completion of the COCM Africa, 2022. (TIF)

S12 Fig The pooled odds ratio of the association between distance to nearest health facility and the completion of the COCM in Africa, 2022. (TIF)

S13 Fig The pooled odds ratio of the association between mass media exposure and the completion of the COCM Africa, 2022. (TIF)

S14 Fig The pooled odds ratio of the association between knowledge regarding pregnancy danger signs and the completion of the COCM in Africa, 2022. (TIF)

S15 Fig The pooled odds ratio of the association between birth preparedness and complication redness plan and the completion of the COCM in Africa, 2022. (TIF)

S16 Fig The pooled odds ratio of the association between maternal autonomy and the completion of the COCM in Africa, 2022. (TIF)

## Abbreviations

ANC: antenatal care
BPCR: birth preparedness and complication readiness
CI: confidence interval
COCM: continuum of care for maternal health
EDHS: Ethiopia demographic and health survey
LMICs: low- and middle-income countries
LPDR: Lao People’s Democratic Republic
NGO: nongovernmental organization
PNC: postnatal care
OR: odds ratio
SBA: skilled birth attendance
SSA: sub-Saharan Africa
