# Supplementary material for: The continuum of care for maternal health in Africa: A systematic review and meta-analysis
Source: PLoS One. 2024 Jul 18;19(7):e0305780. doi: 10.1371/journal.pone.0305780 (PMC11257265; doi:10.1371/journal.pone.0305780)
Supplement: S2 File — (DOCX) [file pone.0305780.s002.docx]

| **PubMed search history** | | | | |
| --- | --- | --- | --- | --- |
|  | Concept | | Search detail | Result |
| Concept 1 map | Maternity continuum of care | 1. Key word | "Maternity continuum of care"[Text Word] OR "continu*"[Text Word] OR "maternity care"[Text Word] OR "Continuum of care"[Text Word] OR "Continuum of care for maternal health services"[Text Word] OR "maternal health*"[Text Word] OR "maternal healthcare services"[Text Word] | 1,292,659 |
|  |  | 1. Mesh term | "Continuity of Patient Care"[MeSH Terms] OR "Maternal Health"[MeSH Terms] OR "Maternal Health Services"[MeSH Terms] | 338,030 |
| Concept 2 map | Level | 1. Key word | "Level"[Text Word] OR "coverage"[Text Word] OR "Magnitude"[Text Word] | 2,741,737 |
|  |  | 1. Mesh term |  |  |
| Concept 3 map | Associated factors | 1. Key word | "Associated factors"[Text Word] OR "determinants"[Text Word] OR "Predictors"[Text Word] | 497,115 |
|  |  | 1. Mesh term |  |  |
| Concept | Women | 1. Key word | "Women"[Text Word] OR "women*"[Text Word] OR "Mother"[Text Word] OR "mother*"[Text Word] | 1,359,052 |
|  |  | 1. Mesh term | "Women"[MeSH Terms] OR "Mothers"[MeSH Terms] | 94,077 |
| Concept 4 map | Africa | 1. Key word | Africa [tw] | 187,104 |
|  |  | 1. Mesh term | “Africa”[Mesh] | 310,430 |
| (("Level"[Text Word] OR "coverage"[Text Word] OR "Magnitude"[Text Word]) AND ("Maternity continuum of care"[Text Word] OR "continu*"[Text Word] OR "maternity care"[Text Word] OR "Continuum of care"[Text Word] OR "Continuum of care for maternal health services"[Text Word] OR "maternal health*"[Text Word] OR "maternal healthcare services"[Text Word] OR ("Continuity of Patient Care"[MeSH Terms] OR "Maternal Health"[MeSH Terms] OR "Maternal Health Services"[MeSH Terms])) AND ("Associated factors"[Text Word] OR "determinants"[Text Word] OR "Predictors"[Text Word]) AND ("Women"[Text Word] OR "women*"[Text Word] OR "Mother"[Text Word] OR "mother*"[Text Word] OR ("Women"[MeSH Terms] OR "Mothers"[MeSH Terms])) AND ("Africa"[Text Word] OR "Africa"[MeSH Terms])) AND ((ffrft[Filter]) AND (humans[Filter]) AND (female[Filter]) AND (medline[Filter]) AND (2015/9/28:2022/9/28[pdat]) AND (english[Filter])) | | | | 586 |
| Hinari Search History | | | |  |
| (\("Maternity continuum of care" OR continu* OR "maternity care" OR "Continuum of care" OR "Continuum of care for maternal health services" OR "maternal health*" OR "maternal healthcare services"\) AND \(Level OR coverage OR Magnitude\) AND \(Women OR "women*" OR Mothers OR mother*\) AND \("Associated factors" OR "determinants" OR "Predictors"[\) AND \(Africa\))** Filter Applied(Full text online, journal article, public health, medicine and nursing, maternal and women health and English, customized date:28/9/2015-28/9/2022) | | | | 283 |
| **Google Scholar** | | | |  |
| Continuum of care for the maternal health in Africa | | | | 132 |

| **Cochrane Search results** | | |
| --- | --- | --- |
| ID | Search Hits | **Results** |
| #1 | "Maternity continuum of care" OR "maternity care" OR "Continuum of care" OR "Continuum of care for maternal health services" OR "maternal healthcare services" | 624 |
| #2 ("Associated factors" OR "determinants" OR "Predictors"):ti,ab,kw (Word variations have been searched) 304637 ("Associated factors" OR "determinants" OR "Predictors"):ti,ab,kw (Word variations have been searched) 304637 | ("Associated factors" OR "determinants" OR "Predictors"):ti,ab,kw (Word variations have been searched) | 304637 |
| #3 | ("Level" OR "coverage" OR "Magnitude"):ti,ab,kw (Word variations have been searched) | 414041 |
| #4 ("Level" OR "coverage" OR "Magnitude"):ti,ab,kw (Word variations have been searched) 414041 | ("Women" OR "women*" OR "Mother" OR "mother*"):ti,ab,kw (Word variations have been searched) | 185331 |
| #5 | (Africa):ti,ab,kw (Word variations have been searched) | 7984 |
| #6 | MeSH descriptor: [Continuity of Patient Care] explode all trees | 29286 |
| #7 | MeSH descriptor: [Maternal Health] explode all trees | 78 |
| #8 | MeSH descriptor: [Maternal Health Services] explode all trees | 2543 |
| #9 | MeSH descriptor: [Africa] explode all trees | 8303 |
| #10 | MeSH descriptor: [Women] explode all trees | 881 |
| #11 | MeSH descriptor: [Mothers] explode all trees | 2238 |
| #12 | MeSH descriptor: [Africa] explode all trees | 8303 |
| #13 | #6 OR #7 OR #8 | 31739 |
| #14 | #10 OR #11 | 3077 |
| #15 | #1 OR #13 | 32225 |
| #16 | #4 OR #14 | 185331 |
| #17 | #5 OR #12 | 13583 |
| #17 | #3 AND #15 AND #3 AND #16 AND #17 | 125 |

| **EMBASE search result** | | |
| --- | --- | --- |
| **No.** | **Query** | **Results** |
| #24. | #15 AND #16 AND #17 AND #18 AND #22 AND [english]/lim AND [female]/lim AND [2015-2022]/py | 421 |
| #23. | #15 AND #16 AND #17 AND #18 AND #22 | 517 |
| #22. | #3 OR #21 | 670,707 |
| #21. | #19 OR #20 | 24 |
| #20. | 'predictors'/exp | 13 |
| #19. | 'determinants'/exp | 11 |
| #18. | #2 OR #14 | 3,583,622 |
| #17. | #1 OR #13 | 1,653,194 |
| #16. | #4 OR #12 | 1,694,586 |
| #15. | #5 OR #11 | 453,181 |
| #14. | #9 OR #10 | 27 |
| #13. | #6 OR #7 OR #8 | 57,372 |
| #12. | 'mother'/exp | 177,580 |
| #11. | 'africa'/exp | 411,359 |
| #10. | 'magnitude'/exp | 17 |
| #9. | 'coverage'/exp | 10 |
| #8. | 'continuum of care'/exp | 24 |
| #7. | 'maternal health service'/exp | 2,643 |
| #6. | 'maternal care'/exp | 55,136 |
| #5. | africa:ti,ab | 162,227 |
| #4. | 'women':ti,ab OR 'women*':ti,ab OR 'mother':ti,ab OR 'mother*':ti,ab | 1,662,072 |
| #3 | 'associated factors':ti,ab OR 'determinants':ti,ab OR 'predictors':ti,ab | 670,701 |
| #2 | 'level':ti,ab OR 'coverage':ti,ab OR 'magnitude':ti,ab | 3,583,610 |
| #1 | 'maternity continuum of care':ti,ab OR continu*:ti,ab OR 'maternity care':ti,ab OR 'continuum of care':ti,ab OR 'continuum of care for maternal health services':ti,ab OR 'maternal healthcare services':ti,ab | 1,605,948 |

**CINHAL**

| Search ID# | Search terms | Result |
| --- | --- | --- |
| \| S1 \| \| --- \| | "Maternity continuum of care" OR "continu*" OR "maternity care" OR "Continuum of care" OR "Continuum of care for maternal health services" OR "maternal health*" OR "maternal healthcare services" | 444,707 |
| S2 | "Associated factors" OR "determinants" OR "Predictors" | 164,163 |
| S3 | "Women" OR "women*" OR "Mother" OR "mother*" | 548,642 |
| S4 | Africa | 53,549 |
| S5 | (MH "Continuity of Patient Care+") | 21,472 |
| S6 | (MH "Maternal Health Services+") | 35,812 |
| S7 | (MH "Women+") | 37,143 |
| S8 | (MH "Mothers+" | 52,251 |
| S9 | (MH "Africa+") | 96,885 |
| S10 | S5 OR S6 | 56,512 |
| S11 | S7 OR S8 | 86,944 |
| S12 | S4 OR S9 | 107,511 |
| S13 | S1 OR S10 | 470,107 |
| S14 | "Level" OR "coverage" OR "Magnitude" | 519,886 |
| S15 | S3 OR S11 | 550,407 |
| S16 | S4 OR S9 | 107,511 |
| S12 | S2 AND S13 AND S14 AND S15 AND S16 | 300 |
| **Limiters - Peer Reviewed; Published Date: 20150901-20220931; English Language; Sex: Female; Language: English** | | |
