# Supplementary material for: The continuum of care for maternal health in Africa: A systematic review and meta-analysis
Source: PLoS One. 2024 Jul 18;19(7):e0305780. doi: 10.1371/journal.pone.0305780 (PMC11257265; doi:10.1371/journal.pone.0305780)
Supplement: S5 File — (DOCX) [file pone.0305780.s005.docx]

**S2 file. Quality appraisal of included study**

**JBI Critical Appraisal Checklist for Analytical Cross Sectional Studies**

| Included studies | **Eight JBI Critical Appraisal Checklist for Cross Sectional Studies(Yes,No,Unclear)**  **If Yes(>=50%).....low risk(two raters).....>1=Yes 0=No/Unclear** | | | | | | | | | | | | | | | | |
| --- | --- | --- | --- | --- | --- | --- | --- | --- | --- | --- | --- | --- | --- | --- | --- | --- | --- |
|  | Q1 | | Q2 | | Q3 | | Q4 | | Q5 | | Q6 | | Q7 | | Q8 | | Over all appraisal |
|  | R1 | R2 | R1 | R2 | R1 | R2 | R1 | R2 | R1 | R2 | R1 | R2 | R1 | R2 | R1 | R2 |  |
| 1. Abebe et al 2022 | Y | Y | Y | Y | Y | Y | Y | Y | Y | Y | Y | Y | Y | Y | Y | Y | 8 |
| 1. Abdo et al 2022 | Y | Y | Y | Y | Y | Y | Y | Y | Y | Y | Y | Y | Y | Y | Y | Y | 8 |
| 1. Arunda et al 2021 | Y | Y | Y | Y | Y | Y | Y | Y | Y | Y | Y | Y | Y | Y | Y | Y | 8 |
| 1. Atnafu et al 2020 | Y | Y | Y | Y | Y | Y | Y | Y | U | U | Y | Y | Y | Y | Y | Y | 7 |
| 1. Camara et al 2021 | Y | Y | Y | Y | Y | Y | Y | Y | Y | Y | Y | Y | Y | Y | Y | Y | 8 |
| 1. Chaka 2022 | Y | Y | Y | Y | Y | Y | Y | Y | Y | Y | Y | Y | Y | Y | Y | Y | 8 |
| 1. Cherie et al 2021 | Y | Y | Y | Y | Y | Y | Y | Y | Y | Y | Y | Y | Y | Y | Y | Y | 8 |
| 1. Dadi et al 2021 | Y | Y | Y | Y | Y | Y | Y | Y | U | U | Y | Y | Y | Y | Y | Y | 7 |
| 1. Dasta et al 2022 | Y | Y | Y | Y | Y | Y | Y | Y | U | Y | U | U | Y | Y | Y | Y | 7.25 |
| 1. Emiru et al 2020 | Y | Y | Y | Y | Y | Y | Y | Y | Y | Y | Y | Y | Y | Y | Y | Y | 8 |
| 1. Haile et al 2020 | Y | Y | Y | Y | Y | Y | Y | Y | Y | Y | Y | Y | Y | Y | Y | Y | 8 |
| 1. H/Mariam et al 2022 | Y | Y | Y | Y | Y | Y | Y | Y | Y | Y | Y | Y | Y | Y | Y | Y | 8 |
| 1. Hamed et al 2018 | Y | Y | U | U | Y | Y | Y | Y | Y | Y | Y | Y | Y | Y | Y | Y | 7 |
| 1. Mohan et al 2017 | Y | Y | Y | Y | Y | Y | Y | Y | Y | Y | Y | Y | Y | Y | Y | Y | 6 |
| 1. Sertsewold et al 2021 | Y | Y | Y | Y | Y | Y | Y | Y | N | N | Y | Y | Y | Y | Y | Y | 7 |
| 1. Shibanuma et al 2018 Ghana | Y | Y | Y | Y | Y | Y | Y | Y | U | U | Y | Y | Y | Y | Y | Y | 7 |
| 1. Sserwanja et al 2021 | Y | Y | Y | Y | Y | Y | Y | Y | U | U | Y | Y | Y | Y | Y | Y | 7 |
| 1. Sserwanja et al 2022 | Y | Y | Y | Y | Y | Y | Y | Y | U | U | Y | Y | Y | Y | Y | Y | 7 |
| 1. Tiruneh et al 2022 | Y | Y | Y | Y | Y | Y | Y | Y | U | U | Y | Y | Y | Y | Y | Y | 7 |
| 1. Tizazu et al 2021 | Y | Y | Y | Y | Y | Y | Y | Y | Y | Y | Y | Y | Y | Y | Y | Y | 8 |
| 1. Yej 2015 Ghana | Y | Y | Y | y | Y | Y | Y | Y | N | N | Y | Y | Y | Y | Y | Y | 8 |
| 1. Tadese et al 2022 | Y | Y | Y | Y | Y | Y | Y | Y | U | U | Y | Y | Y | Y | Y | Y | 7 |
| 1. Galle et 2022 | Y | Y | Y | Y | Y | Y | Y | Y | U | U | Y | Y | Y | Y | Y | Y | 7 |
