# Supplementary figures and images for: The continuum of care for maternal health in Africa: A systematic review and meta-analysis
Source: PLoS One. 2024 Jul 18;19(7):e0305780. doi: 10.1371/journal.pone.0305780 (PMC11257265; doi:10.1371/journal.pone.0305780)

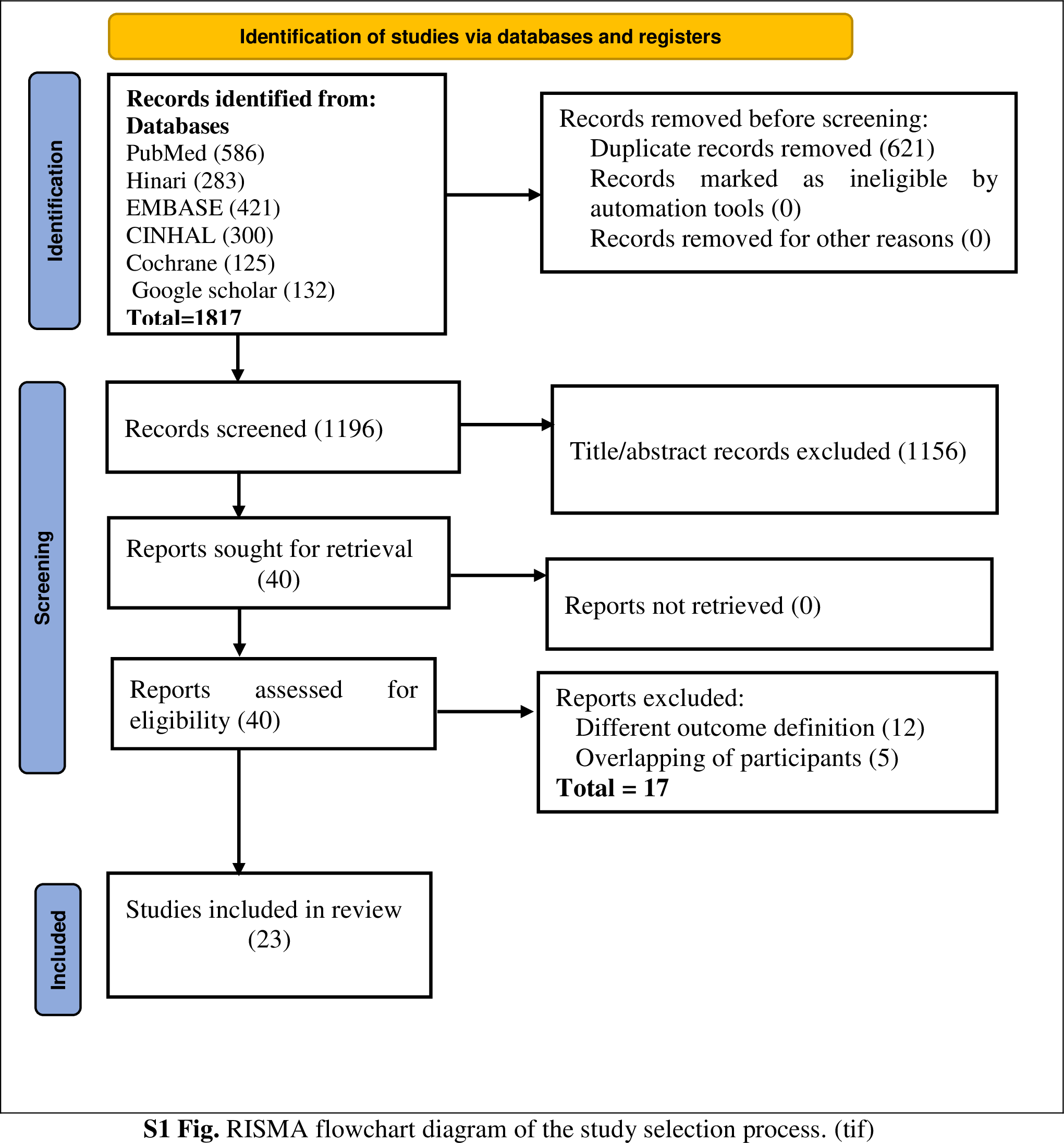

Supplement: S1 Fig — (TIF) [file pone.0305780.s006.tif]

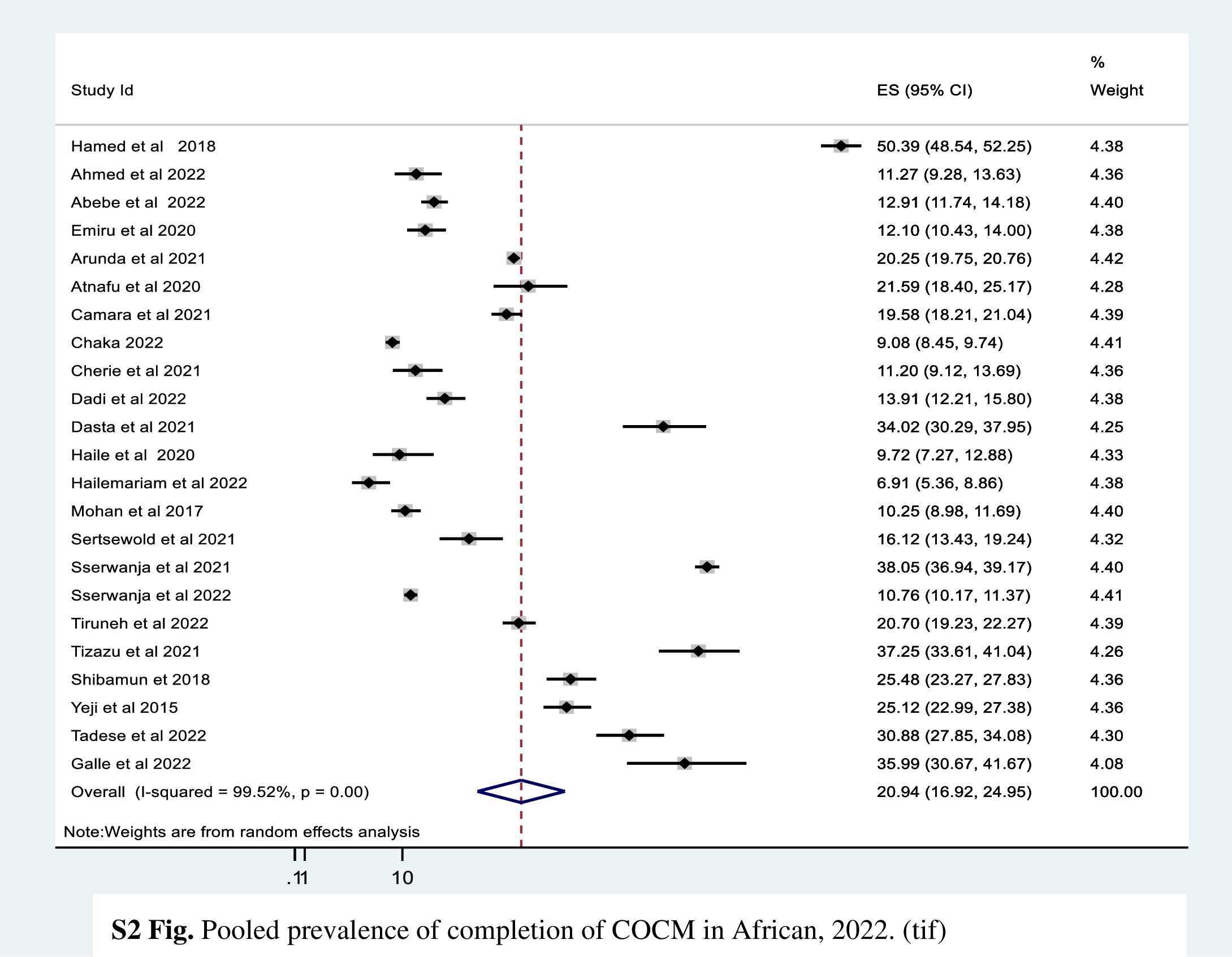

Supplement: S2 Fig — (TIF) [file pone.0305780.s007.tif]

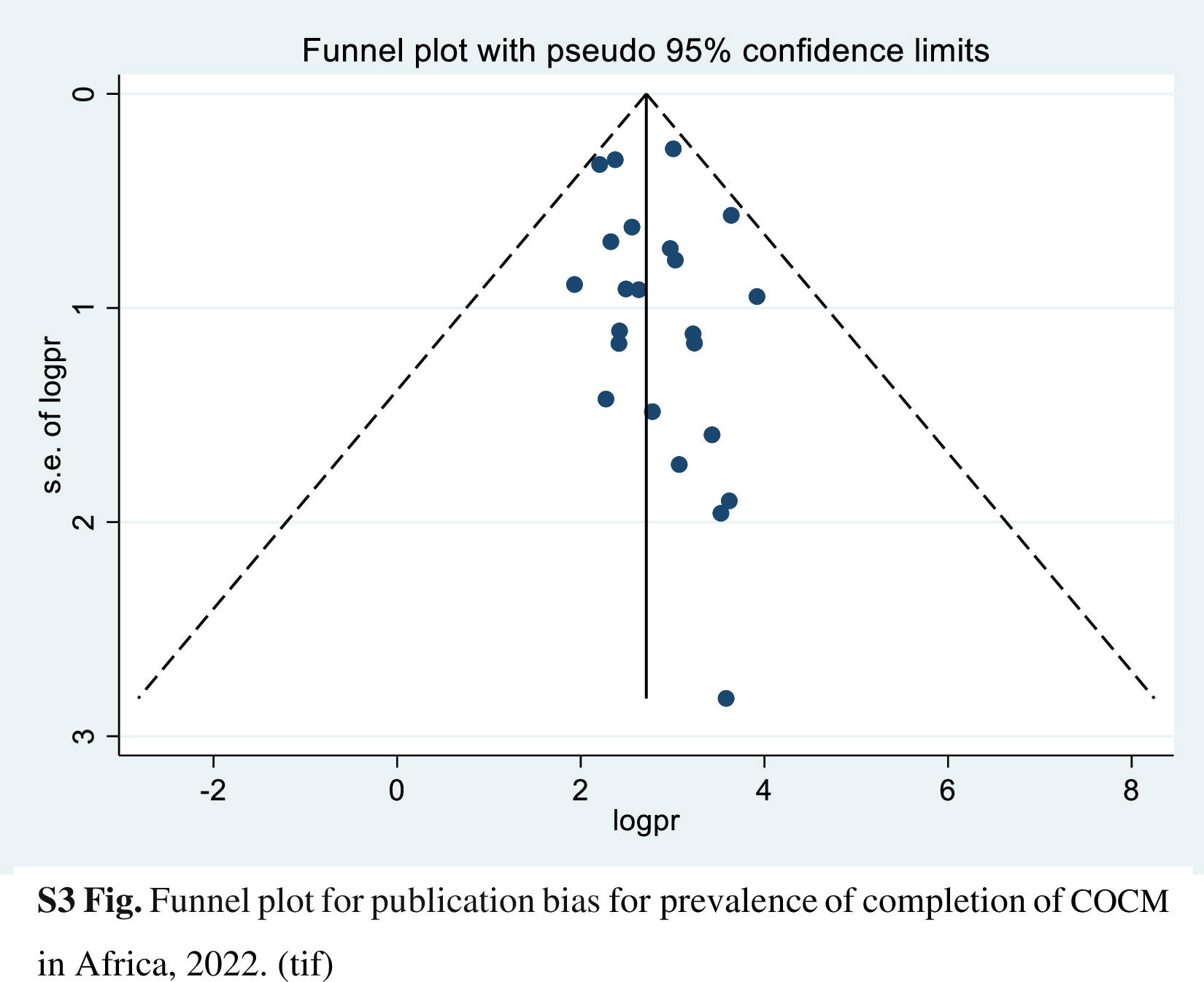

Supplement: S3 Fig — (TIF) [file pone.0305780.s008.tif]

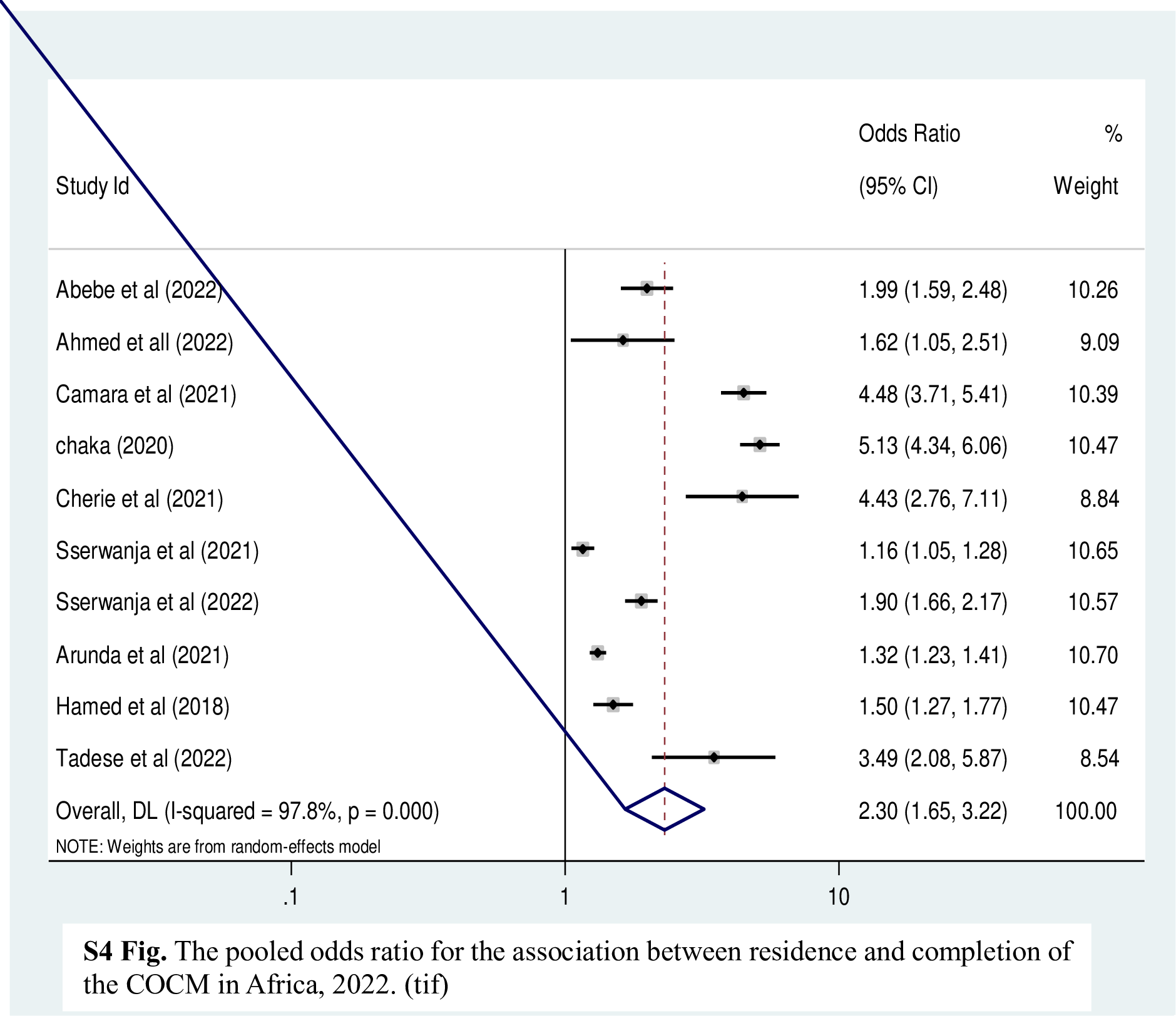

Supplement: S4 Fig — (TIF) [file pone.0305780.s009.tif]

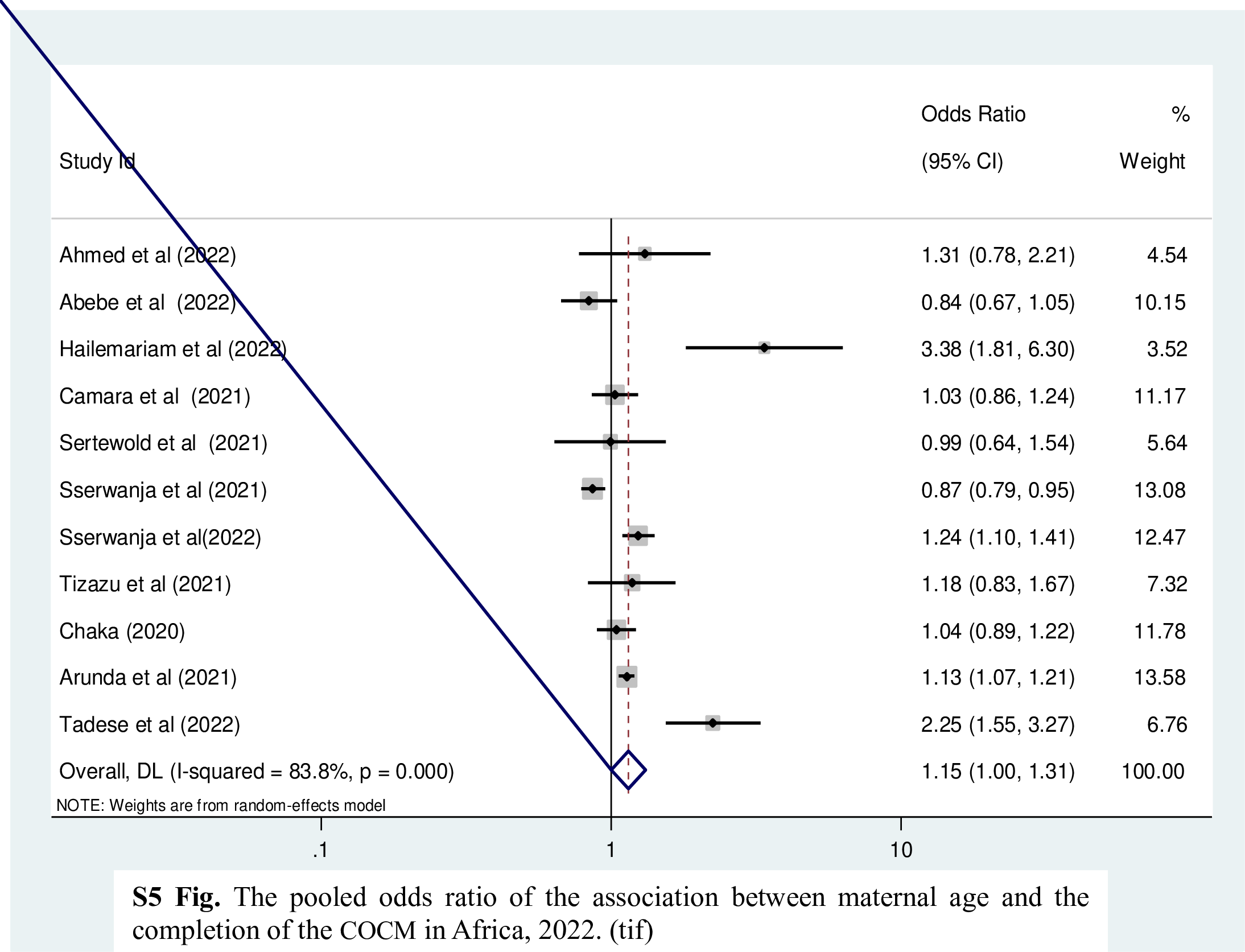

Supplement: S5 Fig — (TIF) [file pone.0305780.s010.tif]

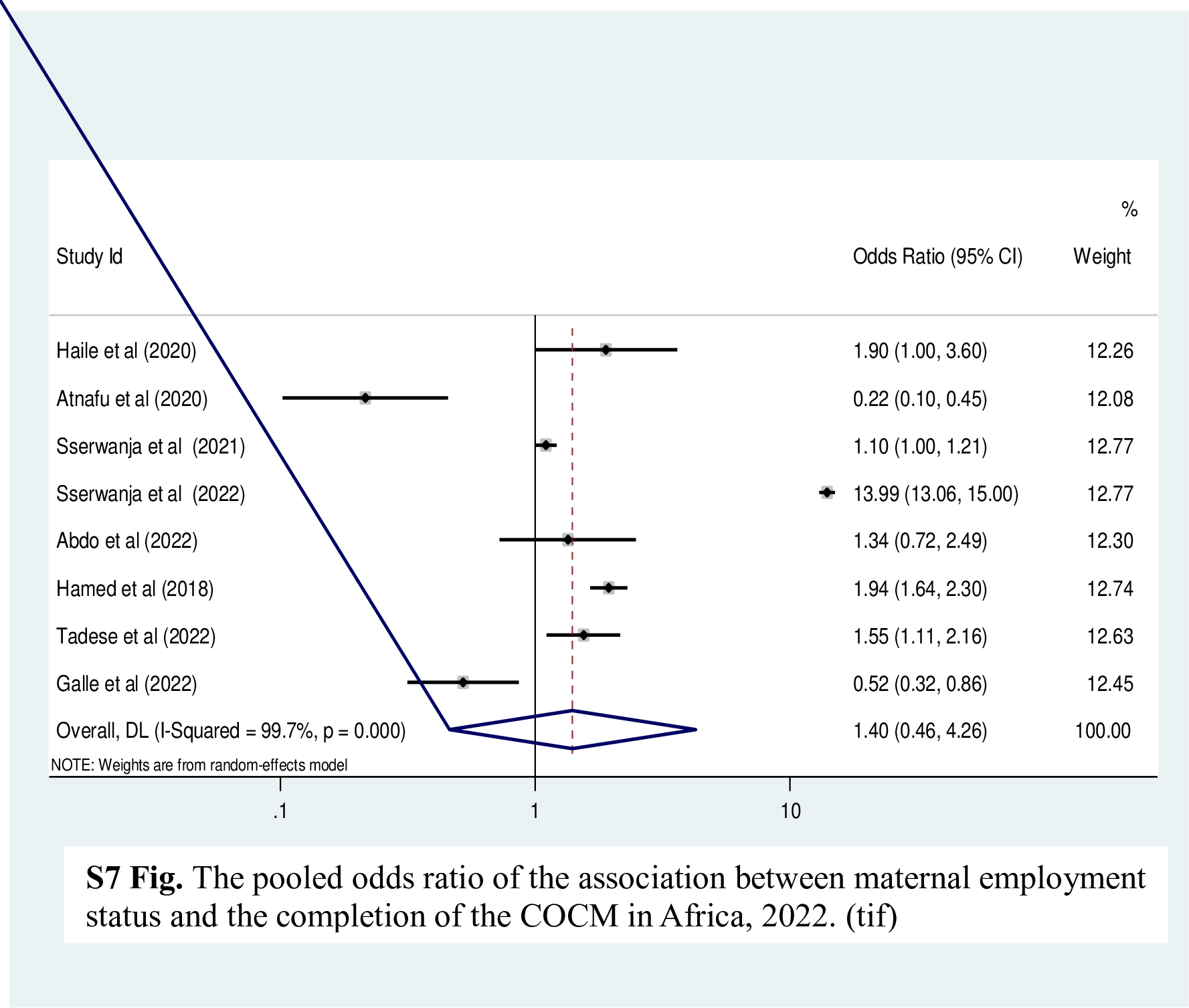

Supplement: S6 Fig — (TIF) [file pone.0305780.s011.tif]

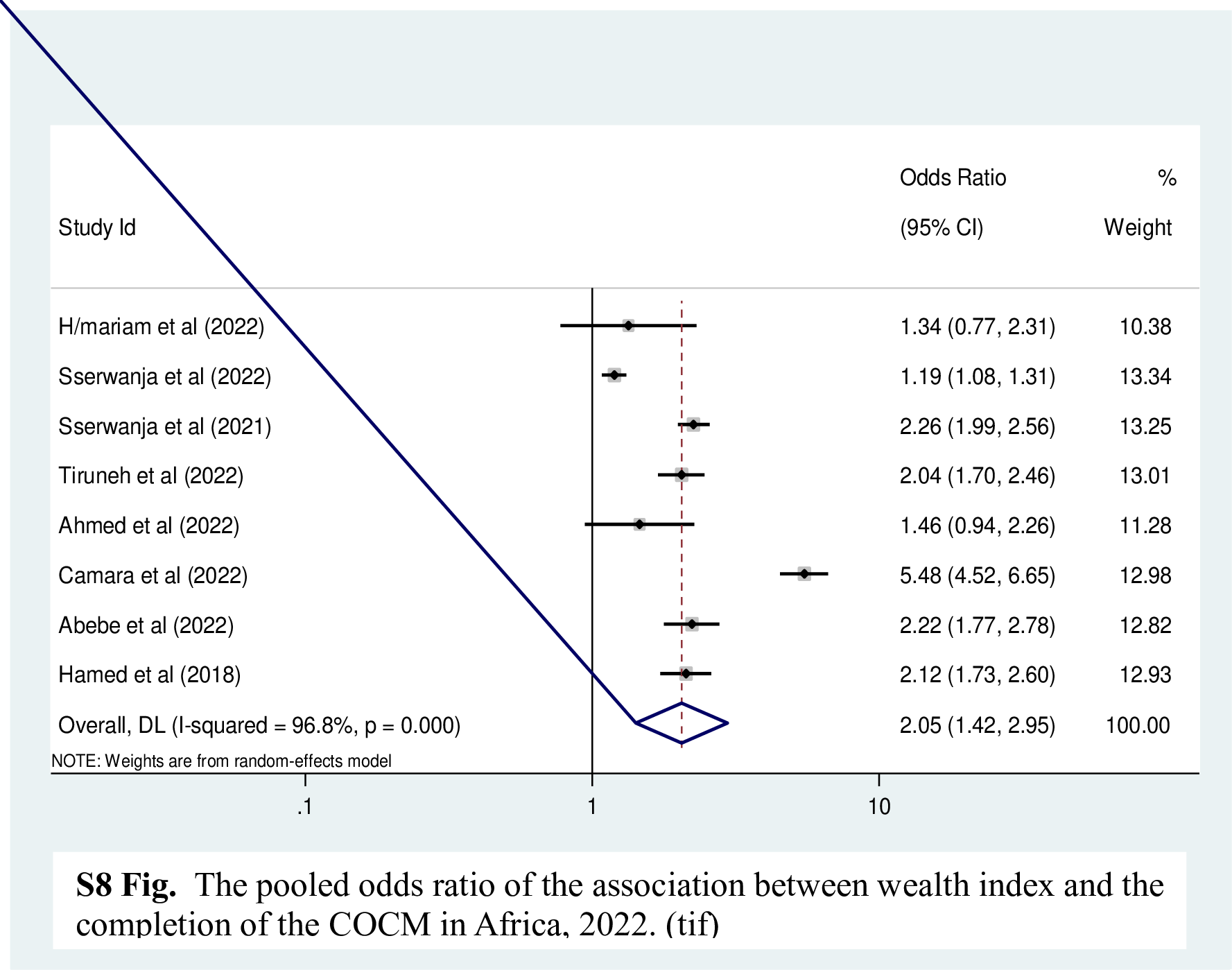

Supplement: S8 Fig — (TIF) [file pone.0305780.s013.tif]

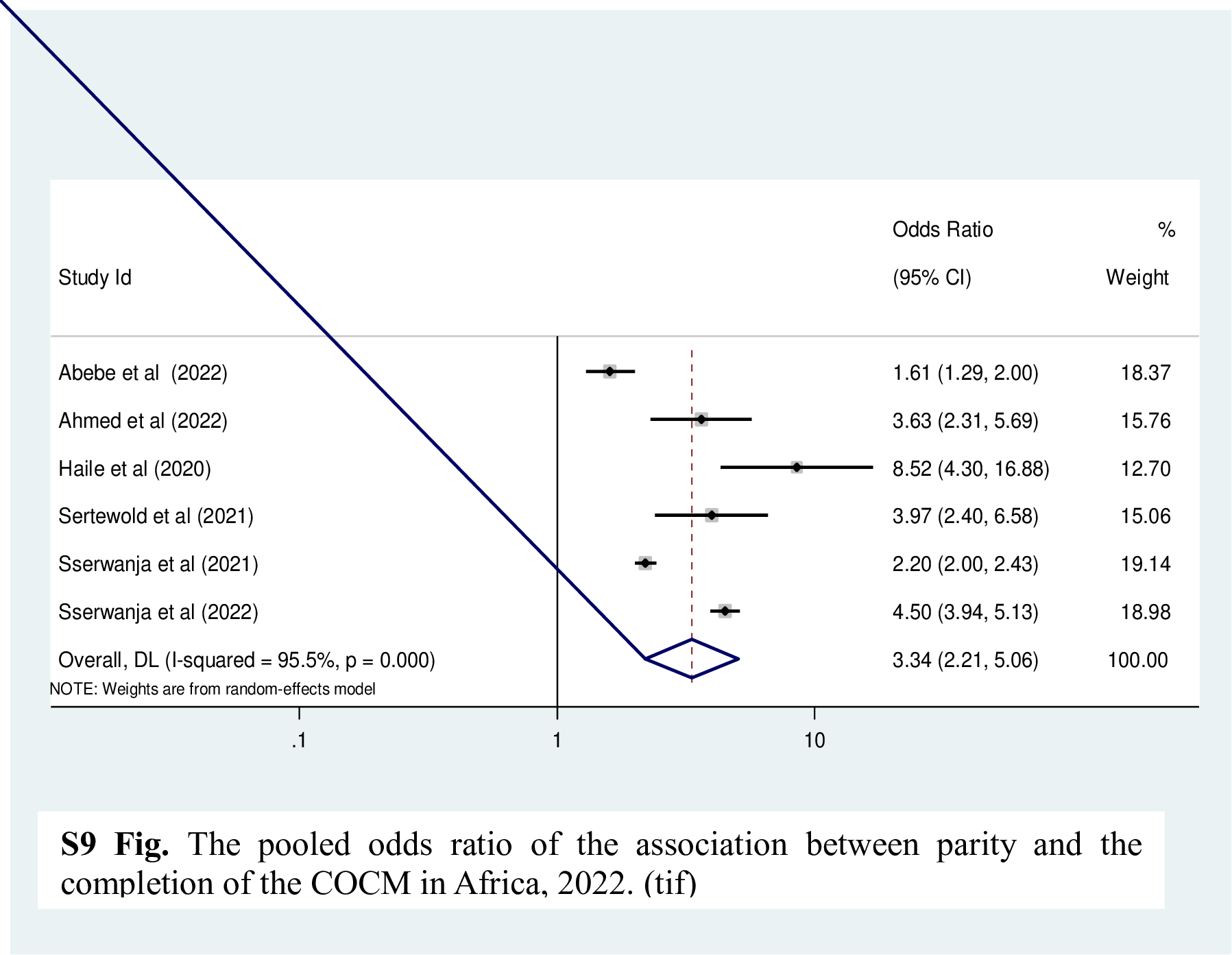

Supplement: S9 Fig — (TIF) [file pone.0305780.s014.tif]

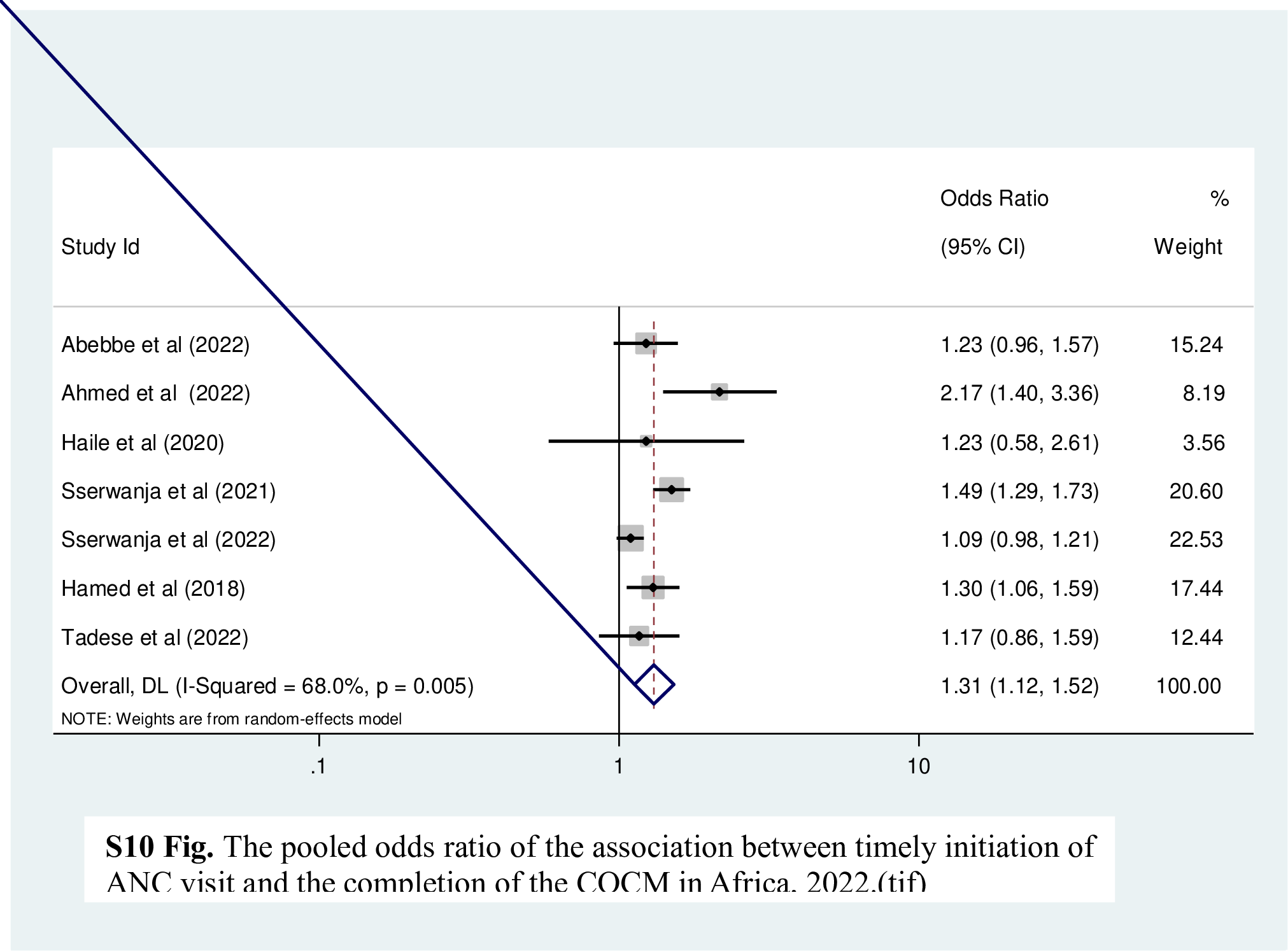

Supplement: S10 Fig — (TIF) [file pone.0305780.s015.tif]

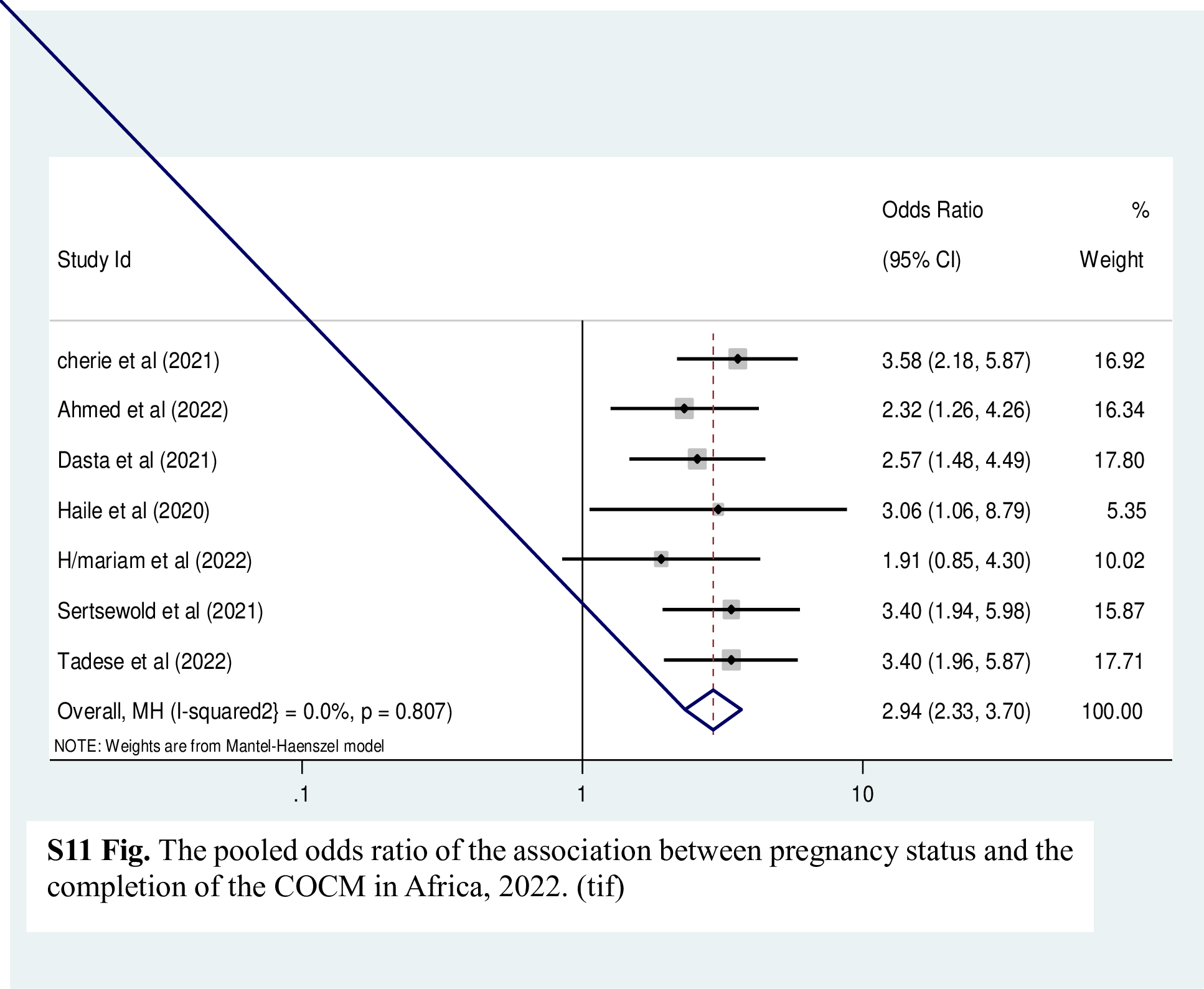

Supplement: S11 Fig — (TIF) [file pone.0305780.s016.tif]

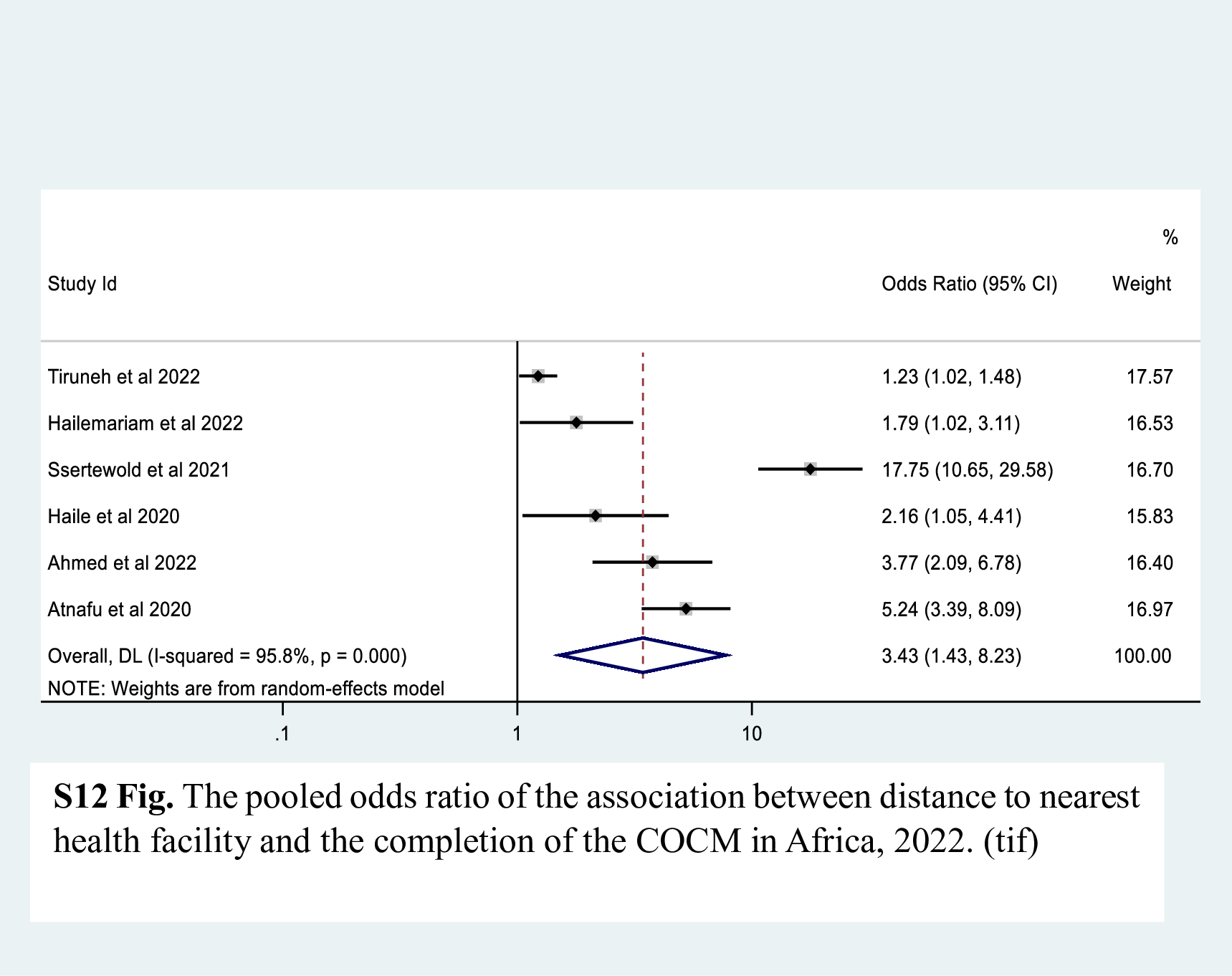

Supplement: S12 Fig — (TIF) [file pone.0305780.s017.tif]

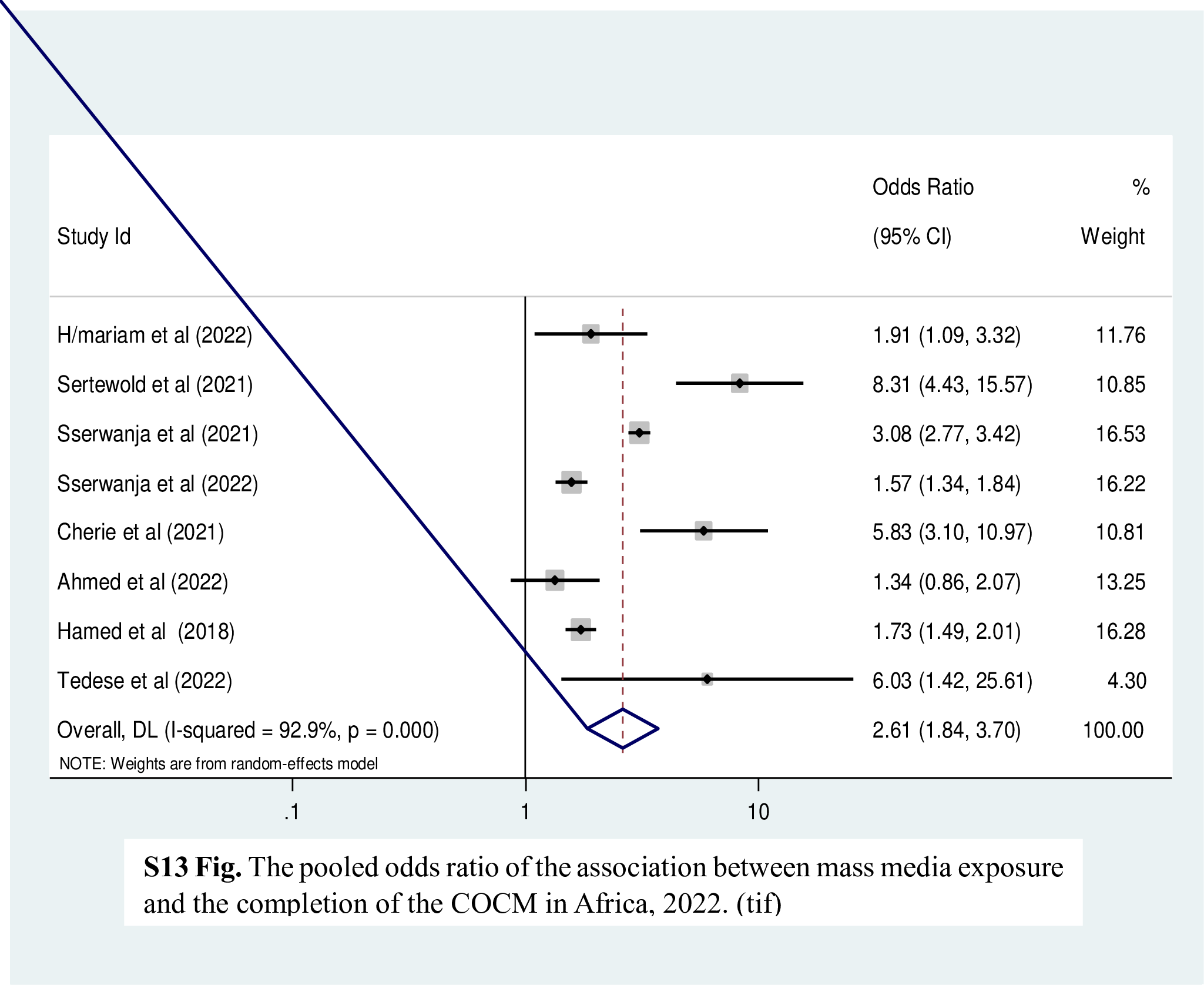

Supplement: S13 Fig — (TIF) [file pone.0305780.s018.tif]

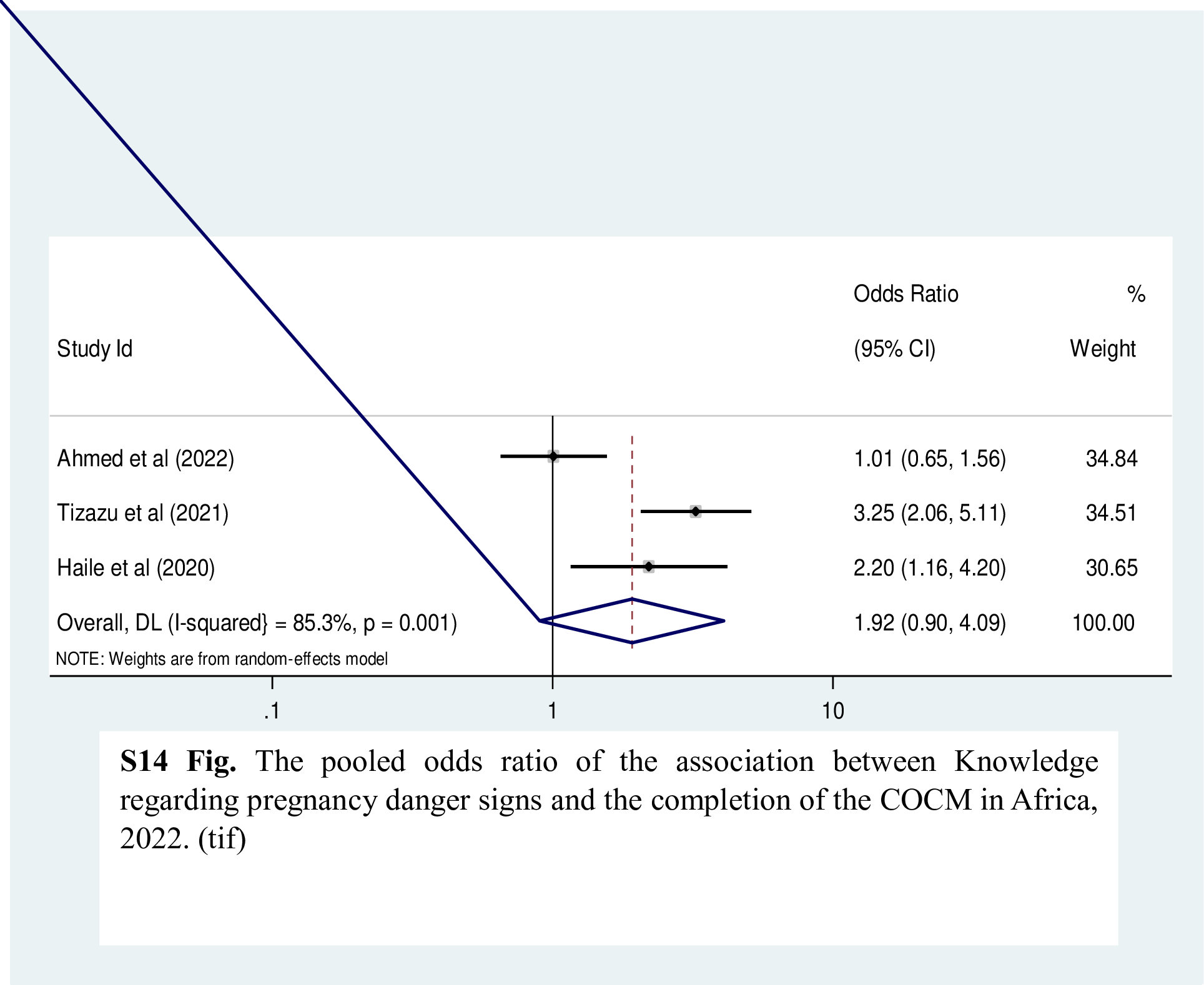

Supplement: S14 Fig — (TIF) [file pone.0305780.s019.tif]

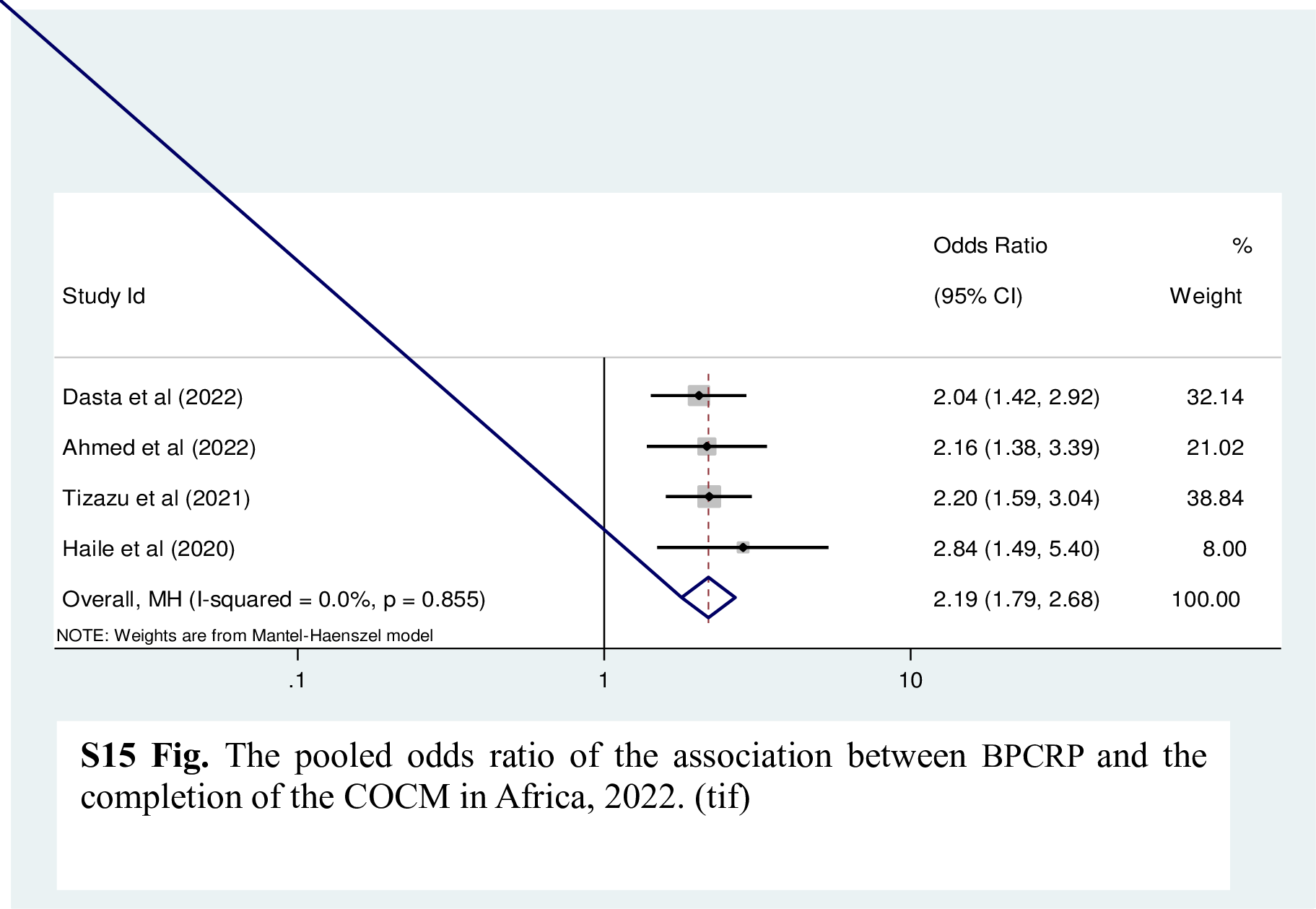

Supplement: S15 Fig — (TIF) [file pone.0305780.s020.tif]

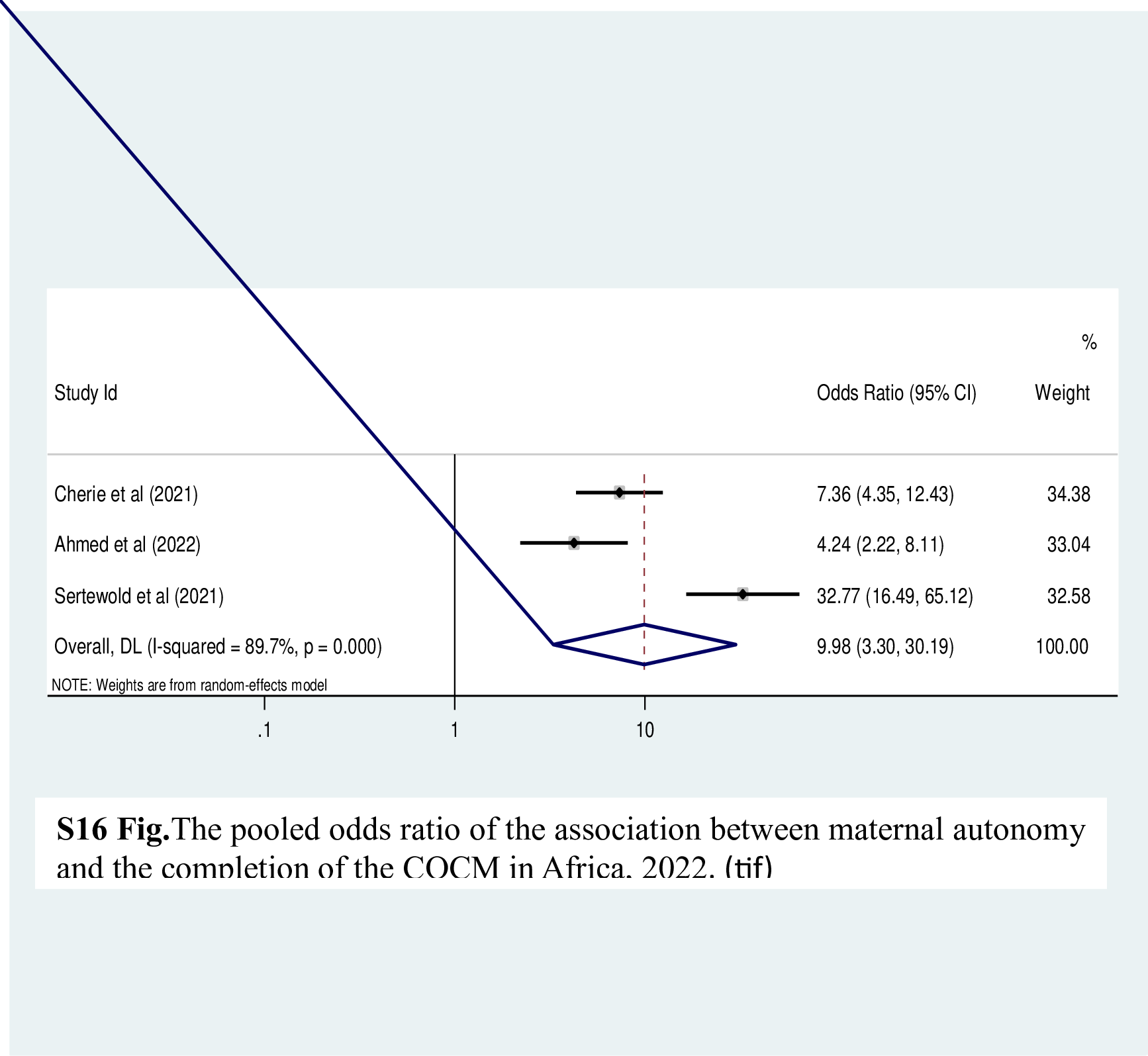

Supplement: S16 Fig — (TIF) [file pone.0305780.s021.tif]
